# Delineating the relationship between stress mindset and primary appraisals: preliminary findings

**DOI:** 10.1186/s40064-016-1937-7

**Published:** 2016-03-15

**Authors:** Christopher J. Kilby, Kerry A. Sherman

**Affiliations:** Centre for Emotional Health, Department of Psychology, Macquarie University, Sydney, NSW Australia; Westmead Breast Cancer Institute, Sydney, NSW Australia

**Keywords:** Stress, Stress mindset, Primary appraisal, Challenge appraisal, Threat appraisal

## Abstract

Stress mindset theory suggests that positive stress beliefs lead to positive, rather than negative, outcomes when engaging with stressors. Similarly, the Transactional Model of Stress predicts that perceiving a stressor as challenging leads to positive outcomes whereas negative perceptions of the stressor as threatening invoke negative outcomes. The aim of this study was to provide preliminary data examining the nature of the relationship between stress mindset and primary appraisals. It was predicted that positive beliefs about stress would be associated with perceiving a stressful situation as more challenging, and inversely related to perceptions of threat. Participants (N = 124) initially completed measures assessing stress mindset, lifetime and current perceived stress, trait anxiety, and self-efficacy. Then participants received a set of instructions regarding a stressful mathematics task, followed by completion of post-manipulation stress mindset and primary appraisals measures, prior to completing the mathematics task. Multiple linear regression analyses revealed that participants who held a greater number of positive beliefs (as opposed to negative beliefs) about stress also perceived the stressor as being more challenging. However, there was no significant relationship between valence of beliefs and threat appraisals. These findings provide initial evidence for the nature of the relationship between valence of stress beliefs and challenge appraisals. Further research is needed to understand how stress beliefs impact on the way in which an individual copes with stressful situations.

## Background

The experience of stress motivates a person to either overcome, withstand, or minimise the demands placed on them by a particular stressor (Salehi et al. 2010). Yet, the impact of this stress can be quite varied, with some benefitting and others suffering as a consequence of their stress (Updegraff and Taylor 2000). There is considerable evidence that stress can produce positive psychological and physiological effects (for a review see Linley and Joseph 2004), however, there is an equally extensive body of evidence associating stress with negative psychological and physiological effects (for a review see Lupien et al. 2007). Yet these two areas of research have been largely explored in isolation of each other (Updegraff and Taylor 2000). As such, understanding why an individual may experience positive or negative effects of a particular stressor remains an important question in stress research that remains largely unanswered.

The Transactional Model of Stress has attempted to address this question (Lazarus and Folkman 1984). The model outlines two processes involved in the stress response: stress appraisal (how an individual perceives a stressor), and coping (how the individual will respond to the stressor; Folkman and Lazarus 1980). Stress appraisal involves an evaluation of both the stressor, known as primary appraisal, and the individual’s perceived available resources for coping with the stressor, known as secondary appraisal (Folkman et al. 1986). Some researchers describe challenge and threat appraisals as a ratio of resources required to cope and resources available to cope, representing an individual’s belief in their own ability to cope with the stressor (e.g., Gallagher 1990; Lyons and Schneider 2005). Others, describe these two appraisals as an evaluation of gains and losses where challenge appraisal evaluates opportunities for success, learning and personal growth and threat appraisal evaluates potential danger to one’s wellbeing or self-esteem (e.g., Blascovich and Mendes 2000; Skinner and Brewer 2002). The present study adopted the latter perspective of gains and losses, as the focus was on the way in which a stressor is perceived.

The Transactional Model of Stress allows for variation in responses to stressors so that it is possible for two people to appraise the same stressor differently whereby one perceives the stressor as highly challenging, and the other as lacking in challenge (Lazarus 2006). An alternative explanation for the subjective nature of primary appraisals that has not yet been investigated is that this may stem from stress mindsets, a cognitive heuristic (Crum et al. 2013). This is defined as a collection beliefs about the positive and negative nature of the stress experience which are thought to collectively influence the way an individual experiences and responds to stressful situations (Crum et al. 2013). There is evidence that individual beliefs motivate an individual to seek out information from their environment that is congruent with that belief (Watson and Tharp 2007). As such, a mindset can be thought of this process en masse, where the mindset influences an individual to initially search for information that aligns with their overarching beliefs about the subject of the mindset. This selective attention to information influences an individual’s actions and responses (Taylor and Gollwitzer 1995). For example, in the context of the intelligence mindsets consisting of fixed and growth mindsets (Dweck 2009), individuals are thought to have a growth mindset if they believe that they are in control of their intelligence, those that do not hold this view are deemed to have a fixed mindset. Individuals with a growth mindset are more likely to perceive and to take up opportunities for learning whereas those with a fixed mindset are less likely to identify and take up these opportunities (Dweck 2009).

Specifically, a stress mindset influences the way information is drawn from stressful situations as a function of whether the individual holds ‘stress-is-debilitating’ or ‘stress-is-enhancing’ beliefs (Crum et al. 2013). If an individual believes that stress is debilitating, their mindset should focus on negative information from stressors that reinforces the negative beliefs, resulting in actions and behaviours that attempt to avoid the stress (Crum et al. 2013). Conversely, individuals who believe stress is enhancing should focus on positive information about stressors that reinforces their beliefs. A stress mindset is described as a continuum from stress-is-debilitating to stress-is-enhancing, whereby it is possible to hold a mix of both enhancing and debilitating beliefs (Crum et al. 2013). When the beliefs are primarily negative, the individual is likely to fixate on the negative aspects of a stressor, and conversely if positive beliefs are predominantly held (Crum et al. 2013). The Stress Mindset Model thus proposes that the way in which we experience stress is related to whether we believe that stress is generally an enhancing or debilitating experience (Crum et al. 2013). Correlational research into the stress mindsets held by employees at an American financial firm has revealed that individuals who have more enhancing stress mindsets report having greater hardiness and resilience to stress, lower levels of perceived stress, and more effective coping strategies and greater performance on stressful work tasks (Crum et al. 2013).

By definition, primary appraisals and stress mindset are similar in that both attempt to explain how we respond to stress through a positive and negative evaluative process (Crum et al. 2013; Lazarus 2006). However, there is one major distinction between these ideas. The primary appraisal process draws heavily on contextual cues (Lazarus and Folkman 1984), whereas stress mindset focuses on stress in general, and disregards the context of the stressor entirely (Crum et al. 2013). While Crum et al. (2013) stipulate that stress mindset and primary appraisals are theoretically distinct this assumption has not yet been empirically investigated. If stress mindset influences the way information about a stressor is sought by the individual, and if primary appraisals are cognitive evaluations of stressors, then it follows that the evaluations made by primary appraisals should be partly dependent on stress mindset. The aim of this study was to apply an experimental paradigm to evaluate the association between stress mindset and primary appraisals. Experimental designs are the optimal way to undertake this type of investigation as it allows for the specific nature of the stressor to be controlled. Experimental control over the stressor allows the researcher to determine when the participants will be exposed to the stressor and allows the researcher to ensure that all participants are exposed to the same stressor. These are favourable characteristics for this study as the context of the stressor is known to influence the stress response (Feinberg and Aiello 2010; Wiemers et al. 2013).

## Method

### Participants and procedure

To achieve the aim of this study, participants’ challenge and threat appraisals were assessed in anticipation of an experimentally administered mathematics stressor. These appraisals were then compared to participants’ stress mindsets. As the stressful mathematics task was completed after reporting on their appraisals, the stressor could not have harmed the participant, and as such, there was no need to address harm/loss appraisals. It was hypothesised that a more enhancing stress mindset would be positively associated with challenge appraisal, and negatively associated with threat appraisal. Power calculations using G*Power (version 3.1.9.2; Faul et al. 2014) estimated that a sample size of 160 participants was required to find a weak significant effect in a multiple regression with a type I error rate of .05 and power of .80. Power calculations were based on a weak effect as there is no research to suggest the expected effect size of this study and because weak effects produce larger sample size requirements.

Study participants were primarily recruited from a pool of undergraduate Psychology students at a university in Sydney, Australia. In total 99 students consented and participated in the study, in exchange for course credit. A further 38 participants were recruited through a snowballing process of community-based contacts of the researchers via social media to increase the total sample size closer to the required 160 to achieved adequate power to detect weak effects. Of these 38 participants, 13 provided consent but did not complete the survey. As such, 25 community-based participants were included in the final analysis. No incentives were offered to community-based participants. All participants were over the age of 18 and reported having no current psychological or stress-related disorders. Approval to conduct this research was granted by the institutional Human Research Ethics Committee.

This study was conducted entirely online, including the consent process. After reading study information and consent forms, participants completed the pre-manipulation questionnaire that assessed stress mindset, demographic characteristics, and a battery of covariate measures including lifetime stress, perceived stress, trait anxiety, and mathematics-related self-efficacy and anxiety, presented in random order. Participants then received instructions for a mathematics stressor task adapted from the Trier Social Stress Test (Kirschbaum et al. 1993). The original Trier Social Stress Test required participants to count backwards out loud from a four-digit number in steps of seven for a period of time. This was modified from the original task such that participants were required to type in their answers online for 10 subtractions (i.e., subtracting seven from 1955 ten times until the participant reached the number 1885). After reading their instructions, participants completed a post-manipulation stress mindset measure and a measure of primary appraisals (both presented in random order) before completing the mathematics task. Participants completed a second stress mindset measure to ensure that the stress mindset measure was not influenced by a stressful situation.

### Measures

#### Stress mindset

The valid and reliable Stress Mindset Measure (Crum et al. 2013) was used to assess stress mindset. Participants indicated their agreement with eight phrases regarding their beliefs about stress, such as “the effects of stress are negative and should be avoided” on a 5-point Likert-type scale (0 “strongly disagree” to 4 “strongly agree”). Mean scores were calculated with a range from 0 to 4 with higher scores representing a greater stress-is-enhancing mindset (i.e., the stress mindset is informed strongly by positive beliefs) while lower scores represent a more debilitating mindset (i.e., the stress mindset is informed strongly by negative beliefs). Acceptable item reliability was achieved in the present study for both pre- (α = .85) and post- (α = .86) manipulation stress mindset.

#### Primary appraisal

Participants’ anticipatory challenge and threat appraisals of the mathematics task were assessed with a reliable and validated measure of cognitive appraisals by Skinner and Brewer (2002). Participants indicated their agreement with 16 statements addressing their thoughts about the mathematics task that they are about to complete, such as “I am focusing on the positive aspects of the mathematics task” (challenge appraisal), and “I worry that I will say or do the wrong thing in the mathematics task” (threat appraisal). Responses were rated on a 6-point Likert-type scale (1 “strongly disagree” to 6 “strongly agree”). Relevant items were averaged to produce total challenge, and total threat appraisal scores (Skinner and Brewer 2002), with higher scores representing a stronger appraisal for that subscale. Acceptable item reliability was achieved in the present study for both challenge (α = .88) and threat (α = .94) appraisal subscales.

### Covariates

A number of stress-related factors were measured and controlled for in multivariate data analyses to reduce possible confounding.

#### Perceived stress

The Perceived Stress Scale (Cohen et al. 1983) is a reliable and validated measure of subjective stress. Participants indicated how often they felt or thought about each scenario described in 14 statements during the past month on a 5-point Likert-type scale (0 “never” to 4 “very often”). For example, “In the last month, how often have you been upset because of something that happened unexpectedly?” Scores were summed with higher scores representing greater perceived stress levels. Acceptable reliability was achieved in the present study for this scale (α = .73).

#### Lifetime stress

Experienced lifetime stress was assessed with the valid and reliable Life Event Inventory (Tennant and Andrews 1976). This scale is comprised of two subscales, Lifestyle Change (the magnitude of stressor-related change in the participant’s life) and Distress (the distressing quality of stressors). Participants endorsed any of the 66 stressors they had experienced (e.g., “Something you valued or cared for greatly was stolen or lost”). Each item had two weightings, one for each subscale. Weightings were summed where greater weightings represented greater distress or change. The current study demonstrated acceptable reliability for each subscale (α’s = .77).

#### Trait anxiety

The State-Trait Anxiety Inventory trait anxiety subscale (Spielberger et al. 1970) is a validated and reliable scale assessing an individual’s habitual, long-term anxiety (Langley et al. 2003). Responses reflect agreement with each of the 20 items (e.g., “I am a steady person”), rated on a 4-point Likert-type scale (1 “Not at all” to 4 “Very much”). Higher total scores represent greater trait anxiety. This study demonstrated acceptable item reliability for trait anxiety (α = .92).

#### General mathematics self-efficacy

Since a mathematics-based stressor was utilised in this study, mathematic self-efficacy was controlled for as it may influence how an individual responds to the mathematics stressor. Due to a lack of general mathematic specific self-efficacy measures, two related measures were adapted for general mathematics to ensure the proper control of mathematic self-efficacy.

##### Mathematics Self-Efficacy and Anxiety Questionnaire

This validated and reliable measure assessed self-efficacy and anxiety concerned with mathematics (May 2009). Items were originally written to be directed towards self-efficacy and anxiety in the mathematics of a calculus class. All items were adjusted to focus on general mathematics ability. The 16 items were on a 5-point Likert-type scale (1 “Never” to 5 “Usually”), such as “I believe I am the kind of person who is good at mathematics”. Items on each subscale were summed for total scores where higher scores represented greater mathematic self-efficacy and lower anxiety. Acceptable reliability was achieved in the present study for both self-efficacy (α = .96), and anxiety (α = .81).

##### Subjective Numeracy Scale

This reliable and valid scale was initially used to assess a medical patient’s understanding of ratios and odds (Fagerlin et al. 2007). Only the 4-item cognitive abilities section of this scale was used in the present study to assess beliefs in coping with certain mathematics tasks (e.g., “How good are you at working with fractions?”). Items were rated on a 6-point Likert-type scale (1 “Not at all good” to 6 “Extremely good”). Scores were summed to generate a total, higher scores represented greater mathematic self-efficacy. Acceptable reliability was achieved in the present study for subjective numeracy (α = .91).

#### Demographic characteristics

Participants provided information about their age, gender, marital status, their highest level of education, and place of residence.

### Statistical analysis

Analyses were performed using SPSS statistical software, Version 22 (IBM 2013), with statistical significance set at *p* < .05 for the main analysis, and *p* < .10 for all analyses selecting covariates for the main analysis. Descriptive statistics were used to describe the obtained sample population. Chi square and *t* test analyses compared the two samples on all measures, and tested the mean difference between pre- and post-manipulation stress mindset to ensure that there was no significant difference these time points (i.e., to provide evidence that stress mindset is independent of the context of a stressor). Any differences between the two samples were controlled for in the main analyses. Pearson’s and Spearman’s correlations were used to assess for multicollinearity (defined as *r* or ρ ≥ 0.8), to check for characteristics related to the outcome variables (challenge appraisal and threat appraisal) that should be treated as covariates in subsequent analyses, and to assess whether pre-manipulation stress mindset scores predicted post-manipulation stress mindset scores. The relationships between stress mindset and both challenge and threat appraisal were assessed with two separate multiple linear regression analyses, one regression for each appraisal scale, controlling for appropriate covariates.

## Results

Descriptive statistics (Table 1) indicated that the majority of the sample were female, under 25, had completed 12 years of schooling, and were drawn from the undergraduate student participant pool. Covariate and outcome descriptives (Table 2) suggested that most participants reported low Life Event Inventory scores, but moderate levels of perceived stress and trait anxiety. In general, the sample reported moderate to high scores for mathematics self-efficacy measures, and low mathematics-related anxiety. Most participants reported low stress mindset scores representing stress-is-debilitating stress mindsets at both pre- and post-manipulation, and reported perceiving the mathematics task as moderately challenging and moderately threatening.

**Table 1:** Descriptives for demographics, outcomes, and covariates by sample source, mean (standard deviation) or N (%)

	Whole sample (N = 124)	Student sample (n = 99)	Community sample (n = 25)	t or χ^2^
Age (*SD*)	21.28 (6.16)	19.66 (4.22)	26.93 (8.30)	−6.30*
Completed high school or higher (%)	123 (96.8 %)	99 (100 %)	24 (85.7 %)	53.58*
Female (vs. male; %)	91 (71.7 %)	40 (64.5 %)	51 (78.5 %)	0.25
Students (vs. community; %)	99 (78.0 %)			

* *p* < .10

**Table 2:** Correlations between all variables

	Mean	SD	1	2	3	4	5	6	7	8	9	10	11	12	13
1. SM1^a^	1.71	0.66													
2. SM2^b^	1.77	0.69	.85**												
3. CA^c^	3.88	0.88	.22**	.32**											
4. TA^d^	3.61	1.12	−.18**	−.16*	−.36**										
5. Life stress^e^	147.44	70.94	.02	.03	.15	.00									
6. Life chng^f^	218.58	99.08	.04	.02	.20**	−.08	.93**								
7. Perc stress^g^	23.24	6.22	−.58**	−.22**	−.34**	.44**	.10	.03							
8. Trait anx^h^	46.28	10.67	−.27**	−.20**	−.35**	.57**	.07	.00	.72**						
9. Math anx^i^	24.83	5.63	.08	.09	.48**	−.47**	−.15*	−.04	−.23**	−.33**					
10. Math SE^j^	21.97	7.12	.04	.06	.42**	−.25**	−.13	−.10	−.15*	−.16*	.64**				
11. Sbj num^k^	16.92	4.96	.09	.14	.48**	−.18**	−.12	−.10	−.05	−.10	.54**	.68**			
12. Age	21.28	6.16	.11	.10	.21**	−.10	.57**	.66**	−.12	−.12	.06	.03	−.05		
13. Educ^l^	2	2−2	.22**	.28**	.15*	−.11	.24**	.32**	−.34**	−.27**	.11	.02	.02	.43**	
14. Gender	1	0–1	.07	.02	−.25**	.22**	−.09	−.13	.12	.13	−.38**	−.23**	−.32**	−.13	.05

Means and standard deviations are displayed for continuous variables only. Median and interquartile range reported for Education and Gender. Education: 0 = some or no schooling, 1 = at least 10 years of schooling, 2 = at least 12 years of schooling, 3 = completed an undergraduate degree, 4 = completed a post-graduate degree, 5 = completed a doctorate degree or higher. Gender: 0 = male, 1 = female. Pearson *r* correlations reported for all correlations except those that include either education or gender. Spearman *ρ* correlations are reported for any correlation containing education or gender

* *p* < .10; ** *p* < .05

^a^SM1 = pre-manipulation stress mindset

^b^SM2 = post-manipulation stress mindset

^c^CA = challenge appraisal

^d^TA = threat appraisal

^e^Life stress = lifetime distress

^f^Life chng = lifetime lifestyle change

^g^Perc stress = perceived stress

^h^Trait anx = trait anxiety

^i^Math anx = mathematic anxiety

^j^Math SE = mathematic self-efficacy

^k^Sbj num = subjective numeracy

^l^Educ = education

The Chi square and *t* test analyses contained in Table 1 revealed that there was a significant difference of age and education between the first year psychology students and community participants whereby first year psychology students were younger and had attained a higher level of education than community members. Given that these samples were combined for later main analyses, age and education were treated as covariates in subsequent analyses.

Bivariate analyses between outcomes, covariates, and demographics are shown in Table 2. Multicollinearity was evident between the two Life Event Inventory subscales; consequently only one measure of life events could be included in further analyses. The Lifetime Adjustment subscale was chosen as it correlated higher with the outcome variables. In regards to the selection of covariates for the main analysis, it was found that trait anxiety, perceived stress, and lifetime adjustment had to be included as covariates with education and gender for the challenge appraisal regression. For the threat appraisal regression, mathematic self-efficacy and anxiety, subjective numeracy, trait anxiety, perceived stress, and age were included as covariates with gender and education. The correlational analysis identified that there was a significant correlation between pre- and post-manipulation stress mindset scores, and a paired samples *t* test found no statistically significant difference between scores at the two time points (Pre-manipulation: *M* = 1.71, SD = 0.66; Post-manipulation: *M* = 1.77, SD = 0.69; *t* = −1.81, *p* = .073). As such, there is evidence to support the assumption that stress mindset, as measured in the present study, does not seem to be influenced by the stressor.

As seen in Table 3, linear regression analyses indicated the expected significant positive relationship between post-manipulation stress mindset and challenge appraisal after controlling for all appropriate covariates. However, as seen in Table 4, there was no significant relationship between post-manipulation stress mindset and threat appraisal after controlling for all appropriate covariates.

**Table 3:** Regression results for the relationship between stress mindset and challenge appraisal

	*β* (SE)	*b*	95 % CI		*Partial* η^2^
			LL	UL	
Stress mindset	.32 (.11)*	.25	.11	.53	.07
Lifetime adjustment	.00 (.00)*	.17	.00	.00	.03
Trait anxiety	−.02 (.01)	−.13	−.03	.00	.02
Perceived stress	−.02 (.02)	−.18	−.05	.01	.01
Education	.04 (.15)	.02	−.26	.34	.00
Gender	−.36 (.16)*	−.19	−.67	−.05	.04

Dependent variable: challenge appraisal. *R* ^2^ = .27. *β* (SE) = unadjusted beta weight (standard error). *b* = adjusted beta weight. *CI* confidence interval, *LL* lower limit, *UL* upper limit. Gender reference group = female

* *p* < .05

**Table 4:** Regression results for the relationship between stress mindset and threat appraisal

	*β* (SE)	*b*	95 % CI		*Partial* η^2^
			LL	UL	
Stress mindset	−.11 (.12)	−.07	−.35	.13	.01
Mathematic SE	−.00 (.02)	−.02	−.04	.03	.00
Mathematic anxiety	−.07 (.02)*	−.33	−.11	−.03	.09
Subjective numeracy	.02 (.02)	.08	−.03	.06	.01
Trait anxiety	.04 (.01)*	.42	.02	.07	.12
Perceived stress	.01 (02)	.07	−.03	.05	.00
Gender	.19 (.20)	.08	−.20	.58	.01
Age	−.00 (.01)	−.02	−.03	.02	.00
Education	.13 (.17)	.06	−.21	.48	.01

Dependent variable: threat appraisal. *R* ^2^ = .44. *β* (SE) = unadjusted beta weight (standard error). *b* = adjusted beta weight. *CI* confidence interval, *LL* lower limit, *UL* upper limit, *Mathematic SE* Mathematic self-efficacy. Gender reference group = female

* *p* < .05

## Discussion

The present study aimed to initiate research and dialogue into the role of stress beliefs in the way we perceive stressful situations; a question of importance given the recent evidence by Crum et al. (2013) that beliefs about stress are related to the way we respond to stressful situations. If beliefs about stress relate to the way we perceive stressful situations, then it may be that beliefs about stress influence the way we respond to stressful situations by altering the way we perceive the situation. This study tested the hypothesis that participants who held predominantly positive beliefs about stress (i.e., an enhancing stress mindset) would perceive the stressor as more challenging and less threatening than those who held predominantly negative beliefs about stress (i.e., a debilitating stress mindset). There was some support for this hypothesis, with participants who held an enhancing stress mindset reporting greater perceptions of challenge than those with a debilitating stress mindset. However, there was no evidence that participants who held an enhancing stress mindset reported lower perceptions of threat than those with a debilitating stress mindset.

The weak positive relationship between stress mindset and challenge appraisal suggests that individuals who believe stress is a positive experience are more likely to perceive the opportunities for gains from a stressful situation. This is in alignment with the hypothesised theory that stress mindset may influence the way in which an individual responds to stressful situations, by first influencing the way the individual perceives the stressful situation. It is known that individuals will adopt different coping styles depending on how challenging and threatening a stressor is perceived to be, as well as whether the individual perceives themselves as having the resources to cope with the stressor (Folkman 2010; Feinberg and Aiello 2010; Jamieson et al. 2013). Along with other variables, it would seem that one determinant of perceptions of challenge may be stress mindset. Further study is required to ascertain the implications of this finding on coping.

While there was a significant and negative correlation between stress mindset and threat appraisal, this relationship was lost after controlling for appropriate covariates, the most influential of which were trait anxiety and anxiety regarding mathematics. It is possible that the lack of a unique relationship between stress mindset and threat appraisal may be due to the stressor being too mild to invoke feelings of potential loss. Given the ethical considerations of inducing stress on human participants, this study utilised a minimally invasive stressor to probe for a possible relationship between stress mindset and primary appraisal. This could have reduced level of threat in the stressor. Stress mindset theory would predict that an individual with a debilitating stress mindset would perceive a greater level of threat in a stressor than an individual with an enhancing stress mindset if there was indeed threatening information to focus on. However, given that there was a bivariate relationship between threat appraisal and stress mindset, it may be that a more potent stressful situation would invoke the hypothesised relationship between stress mindset and threat appraisal. Alternatively, it may be that anxiety, rather than stress mindset, influences the degree to which an individual perceives opportunities for loss in a stressor. There is some research to suggest that trait anxiety focusses an individual’s attention on the negative aspects of a stressor (Eysenck et al. 2007). The fact that the two anxiety constructs related to stress mindset stronger than any other variable in this study offers some support for this notion, however further testing is required to confirm such suspicions.

In interpreting these findings, study limitations need to be considered. The present study demonstrated its findings in the presence of an online minimally invasive acute stressor. While the implications of the stressor being minimally invasive have been discussed it is worth noting the implications of using an acute stressor as opposed to a more enduring stressor. It is possible that the prolonged or repeated exposure to a stressor may alter ones stress mindset, an effect that would not be detectable in a study using an acute stressor. Therefore, future researchers should explore the relationship between stress mindset and both objective and subjective levels of stress over time. Further to this point, by conducting the study entirely online, it was not possible to control the extraneous environment of the study participants. As such, researchers should aim to conduct future stress mindset research in person, rather than online. Furthermore, there is no research on the developmental trajectory of a stress mindset, as such it is unknown at what age an individual begins to develop ideations about stress. However, it is well known that students, both at a secondary and tertiary level, experience high degrees of academic-related stress (Regehr et al. 2013; Galbraith and Brown 2011; Cohen and Khalaila 2014). Given that all participants in the present study had completed a high school education it can be assumed that all participants have experienced some degree of academic stress. Furthermore, all participants in the present study reported perceiving themselves to having experienced some level of stress over the previous month. As such, there is evidence to suggest that the sample in the present study should have developed a stress mindset of some sort.

## Conclusions

Through a comparison of stress mindset with primary appraisals made of a minimally invasive stressful situation, we have provided preliminary evidence that more enhancing stress mindsets may be associated with greater challenge appraisals. Previous research has suggested that greater challenge appraisals are associated with more efficient and beneficial coping behaviours (Folkman 2010). Additionally, Crum et al. (2013) have previously demonstrated that more enhancing stress mindsets are associated with better post-stressor outcomes for both physical and mental health. Together, with the findings of the present study, there is now reason to suspect that stress mindset may influence the way a stressor is perceived, which in turn influences the way in which an individual responds to that stressor. As such, there is now a need to examine the causal role of stress mindset in not only primary appraisals, but also secondary appraisals and coping. Knowing this information may help future research to develop or improve theoretical models and interventions targeted at stress and coping. Such an analysis may reveal the true influence of stress mindset on the stress response. However, readers should be ever reminded that the present study was conducted with a minimally invasive stressor. Yet, given that there is some evidence of a relationship between stress mindset and primary appraisals in such a low impact stressful situation, one would expect that this relationship would only strengthen in the presence of a more potent stressful situation.

## References

[CR004] Blascovich J, Mendes W, Forgas J (2000). Challenge and threat appraisals: the role of affective cues. Feeling and thinking: the role of affect in social cognition.

[CR1] Cohen M, Khalaila R (2014). Saliva pH as a biomarker of exam stress and a predictor of exam performance. J Psychosom Res.

[CR2] Cohen S, Kamarck T, Mermelstein R (1983). A global measure of perceived stress. J Health Soc Behav.

[CR3] Crum AJ, Salovey P, Achor S (2013). Rethinking stress: the role of mindsets in determining the stress response. J Pers Soc Psychol.

[CR4] Dweck C (2009). Mindsets: developing talent through a growth mindset. Olymp Coach.

[CR5] Eysenck M, Derakshan N, Santos R, Calvo M (2007). Anxiety and cognitive performance: attentional control theory. Emotion.

[CR6] Fagerlin A, Zikmund-Fisher BJ, Ubel PA, Jankovic A, Derry HA, Smith DM (2007). Measuring numeracy without a math test: development of the Subjective Numeracy Scale. Med Decis Mak.

[CR7] Faul F, Buchner A, Erdfelder E, Lang A (2014) G*Power (version 3.1.9.2)

[CR8] Feinberg J, Aiello J (2010). The effect of challenge and threat appraisals under evaluative presence. J Appl Soc Psychol.

[CR9] Folkman S, Carr B, Steel J (2010). Stress, coping, and hope. Psychological aspects of cancer.

[CR001] Folkman S, Lazarus RS (1980). An analysis of coping in a middle-aged community sample. J Health Soc Behav.

[CR10] Folkman S, Lazarus RS, Gruen RJ, DeLongis A (1986). Appraisal, coping, health status, and psychological symptoms. J Pers Soc Psychol.

[CR11] Galbraith N, Brown K (2011). Assessing intervention effectiveness for reducing stress in student nurses: quantitative systematic review. J Adv Nurs.

[CR002] Gallagher D (1990). Extraversion, neuroticism and appraisal of stressful academic events. Personal Individ Differ.

[CR12] IBM (2013). Statistical package for the social sciences.

[CR13] Jamieson J, Mendes W, Nock M (2013). Improving acute stress responses: the power of reappraisal. Curr Dir Psychol Sci.

[CR14] Kirschbaum C, Pirke K-M, Hellhammer DH (1993). The ‘Trier Social Stress Test’—a tool for investigating psychobiological stress responses in a laboratory setting. Neuropsychobiology.

[CR15] Langley JM, Halperin SA, Smith B (2003). A pilot study to quantify parental anxiety associated with enrollment of an infant or toddler in a phase III vaccine trial. Vaccine.

[CR16] Lazarus R (2006). Stress and emotion: a new synthesis.

[CR17] Lazarus R, Folkman S (1984). Stress, appraisal, and coping.

[CR18] Linley PA, Joseph S (2004). Positive change following trauma and adversity: a review. J Trauma Stress.

[CR19] Lupien SJ, Maheu F, Tu M, Fiocco A, Schramek TE (2007). The effects of stress and stress hormones on human cognition: implications for the field of brain and cognition. Brain Cogn.

[CR003] Lyons J, Schneider R (2005). The influence of emotional intelligence on performance. Personal Individ Differ.

[CR20] May DK (2009) Mathematics Self-Efficacy and Anxiety Questionnaire. Doctoral dissertation, University of Georgia

[CR21] Regehr C, Glancy D, Pitts A (2013). Interventions to reduce stress in university students: a review and meta-analysis. J Affect Disord.

[CR22] Salehi B, Cordero I, Sandi C (2010). Learning under stress: the inverted-U-shape function revisited. Learn Mem.

[CR23] Skinner N, Brewer N (2002). The dynamics of threat and challenge appraisals prior to stressful achievement events. J Pers Soc Psychol.

[CR24] Spielberger C, Gorsuch RL, Lushene RE (1970). The state-trait anxiety inventory (test manual).

[CR25] Taylor SE, Gollwitzer PM (1995). Effects of mindset on positive illusions. J Pers Soc Psychol.

[CR26] Tennant C, Andrews G (1976). A scale to measure the stress of life events. Australas Psychiatry.

[CR27] Updegraff JA, Taylor SE, Harvey J, Miller E (2000). From vulnerability to growth: positive and negative effects of stressful life events. Loss and trauma: general and close relationship perspectives.

[CR28] Watson D, Tharp R (2007). Self-directed behaviour.

[CR29] Wiemers US, Schoofs D, Wolf OT (2013). A friendly version of the Trier Social Stress Test does not activate the HPA axis in healthy men and women. Stress.

